# Synthesis, biological evaluation of antioxidant-antibacterial activities and computational studies of novel anthracene- and pyrene-based Schiff base derivatives

**DOI:** 10.3906/kim-2005-61

**Published:** 2020-08-18

**Authors:** Ayşegül GÜMÜŞ, Veysi OKUMUŞ, Selçuk GÜMÜŞ

**Affiliations:** 1 Department of Chemistry, Faculty of Science, Van Yüzüncü Yıl University, Van Turkey; 2 Department of Biology, Faculty of Arts and Sciences, Siirt University, Siirt Turkey

**Keywords:** Anthracene, pyrene, Schiff base, antioxidant, antibacterial, electronic properties

## Abstract

Schiff base derivatives with anthracene- and pyrene-based units, **A1-A6** and **P1-P6** were synthesized (89%–99% yields). Schiff base derivatives were designed to possess an heterocyclic moiety on one side to enhance the coordination ability towards metals. To investigate the biological assay of the newly synthesized compounds, their DPPH (2,2-diphenyl-1-picrylhydrazyl) radical scavenging, metal chelating, reducing power, antibacterial and DNA binding activities were tested. **A6** (63.1%) showed the maximum free radical scavenging activity among all. However, compound **P3** at concentration of 200 μg/mL possessed the highest metal chelating (45.8%) activity and power of reduction. In addition, **P3** and **A6** showed antibacterial activity against all bacteria tested and both compounds were very well bound to CT-DNA. Density functional theory method with B3LYP/6-311++G(d,p) basis set was performed to get information about the structural and electronic properties of the present compounds. In addition, the metal coordination properties of the dimers of the parent Schiff bases were investigated through interactions with Zn^2+^.

## 1. Introduction

Schiff bases belong to a sub-group of imines or azomethines. Their exceptional popularity in organic chemistry can be attributed to their easy synthesis techniques from cheap starting materials and air stability as well. Moreover, a wide range of pharmacological and biological applications add their need of synthesis. Each novel Schiff base derivative received a great deal of attention for the imine (-C=N) bond forming the heart of these molecules, which acts a very important role for bioactivity [1,2]. There have been many published reports in the literature revealing remarkable potential of Schiff bases as antibacterial [3], antifungal [4], anticancer [5], urease inhibitor [6], antioxidant [7,8]. Additionally, their antiglycation activities [9] antiinflammatory [10,11], antitumor [12,13], antiviral [14], antipyretic [15], anti-HIV-1 [16], antiproliferative [17,18] abilities have also been reported. In addition, the presence of nitrogen in the core structure lead them to be used as a chemosensor [19–21].

Schiff bases display strong chelating ability due to the presence of unpaired electrons on nitrogen atom of the azomethine moiety, especially when one or more donor atoms located in close neighbourhood to the azomethine group. Schiff bases become interesting ligands for coordination chemistry by their chelating ability together with the convenience of separation and flexibility in changing the structural formation about the C=N group [22].

The potential of Schiff bases in antitumor, antimalarial, antimicrobial, antiviral, antipyretic, antiproliferative, and antineoplastic activity appear to be enhanced by incorporation of a carbon nitrogen double bond with heteroatom rings [23–25].

Combination of new potential biologically active species with pharmacophoric moieties identified and derived from known bioactive molecules has been known for designing novel biologically active compounds [26]. Inspired by the biological profile of Schiff base derivatives containing heteroatom rings and their increasing importance in pharmaceutical and biological science, in this study our goal was to synthesize anthracene- and pyrene-based Schiff base derivatives to obtain certain new chemicals for the intensified biological activities. Chemical units containing anthracene or pyrene framework possess prolonged π-conjugation property, high stability, good quantum yield and influential photoluminescence abilities which lead them to be well suited for potentail chemosensors [27–31]. Prompted by this information, we can say that these anthracene- and pyrene-based Schiff base derivatives are good candidates for chemosensors.

## 2. Results and discussion

### 2.1. Chemistry

Anthracene-based Schiff base derivatives **A1-A6** were synthesized by the reaction of 1-aminoanthracene with aldehyde derivatives (furan-2-carbaldehyde, thiophene-2-carbaldehyde, 3-hydroxypicolinaldehyde, benzofuran-2-carbaldehyde, benzo[b]thiophene-2-carbaldehyde and 8-hydroxyquinoline-2-carbaldehyde) in ethanol by the catalysis of acetic acid (Scheme).

**Scheme:**
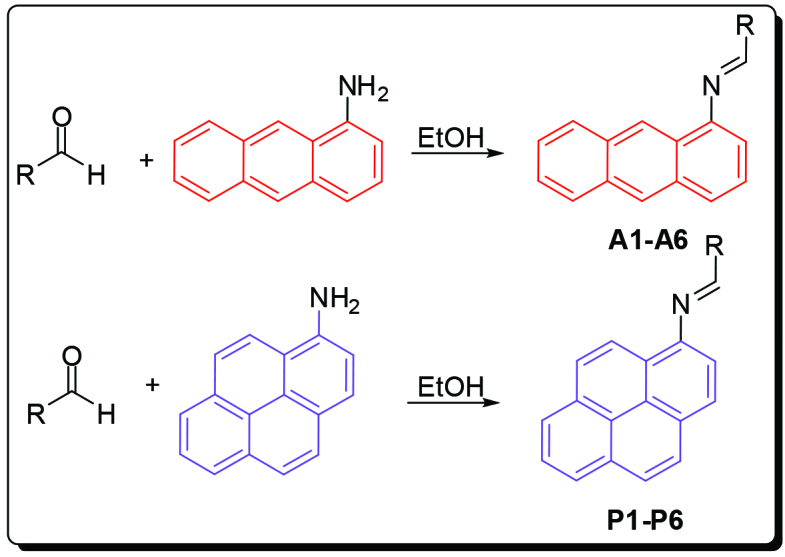
Synthesis of anthracene- and pyrene-based Schiff base derivatives.

The successful synthesis of anthracene-based Schiff base derivatives **A1-A6** prompted us to investigate the construction of pyrene-based Schiff base compounds from aminopyrene. Reactions afforded Schiff bases **P1-P6** in good yields (89%–99%) (Table 1). The synthesis and anticancer activity investigation of **P1** [32], and synthesis and chemosensor application of **P6** [33] have been reported in the literature.

**Table 1:** Results of the Schiff base syntheses.

Entry	Aldehyde	Product	Yield (%)	Entry	Product	Yield (%)
1	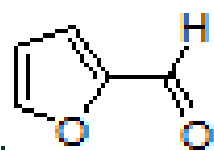	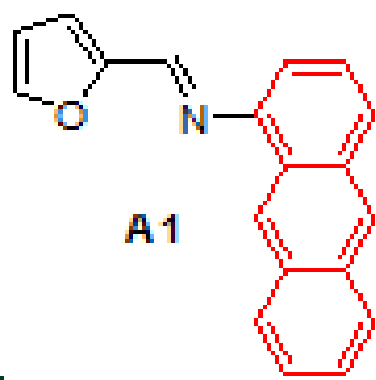	96	7	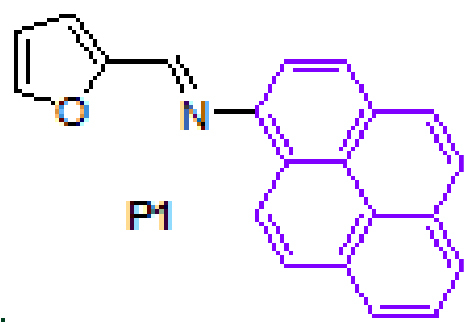	93
2	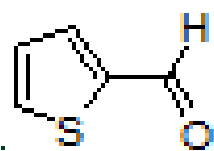	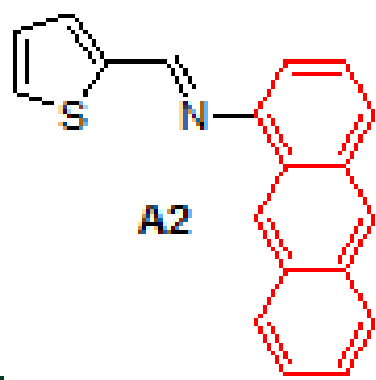	94	8	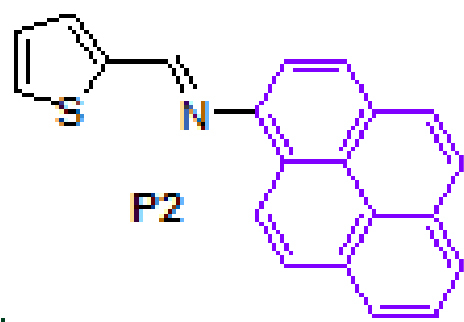	94
3	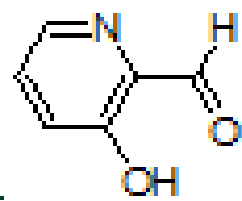	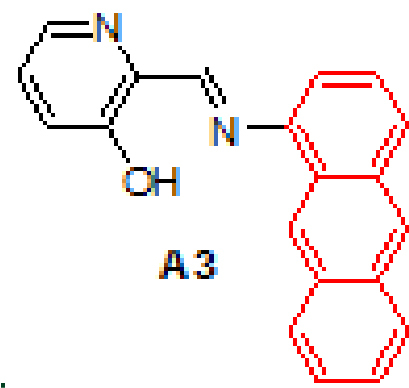	95	9	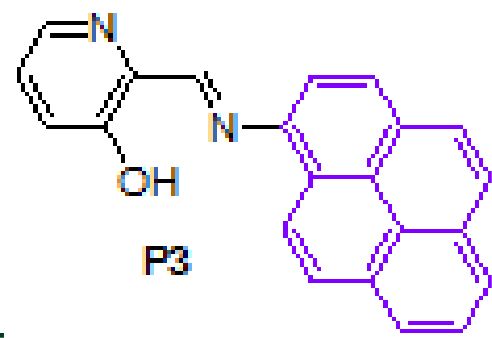	89
4	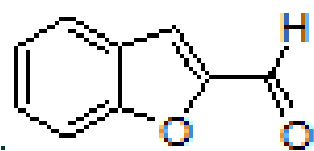	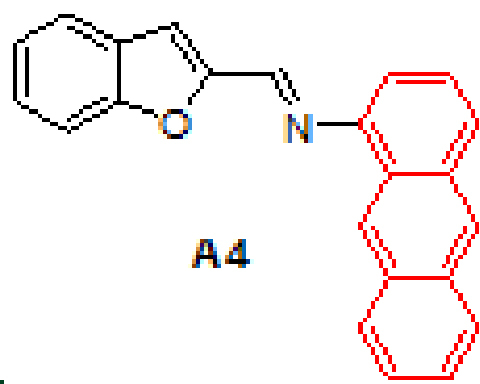	90	10	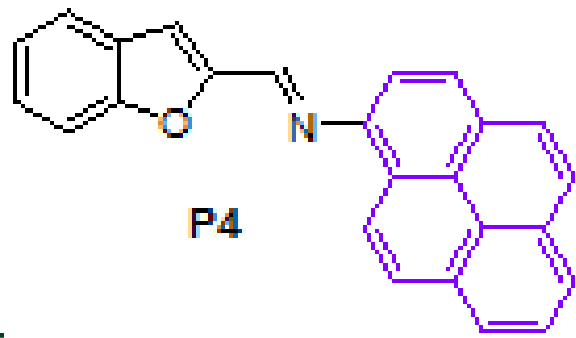	95
5	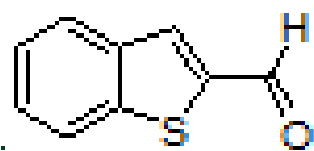	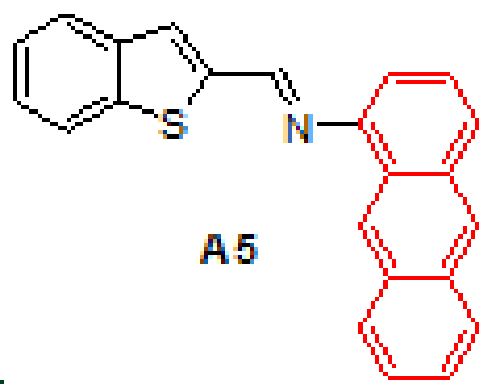	96	11	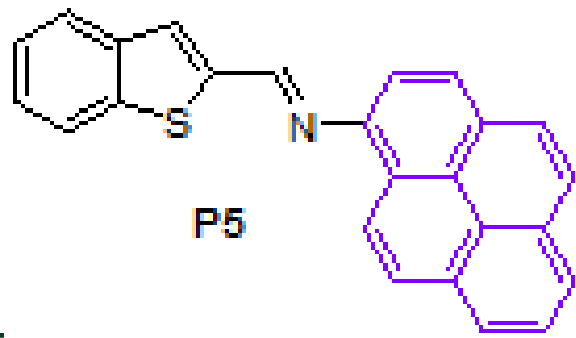	92
6	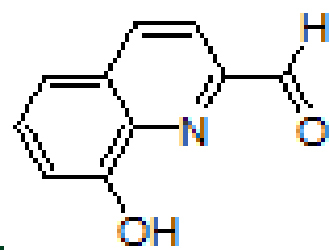	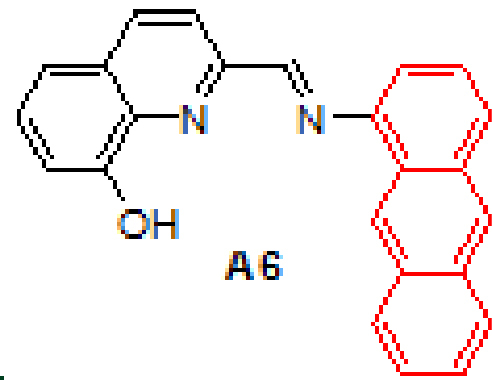	99	12	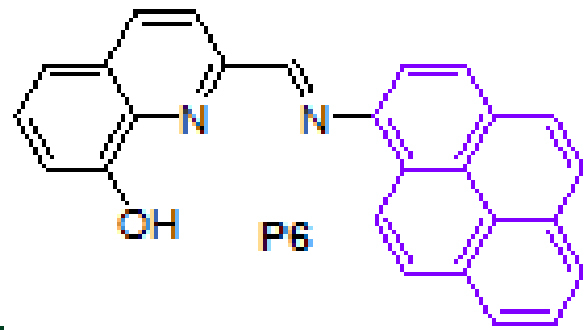	98

### 2.2. Antioxidant activity

**Figure 1:**
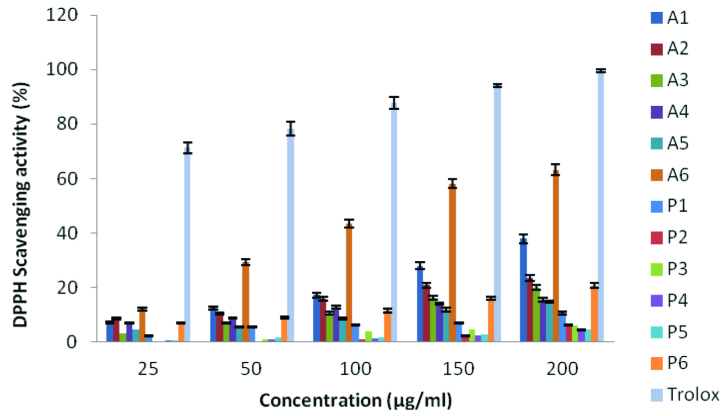
DPPH radical-scavenging activity of compounds.

Figure 1, all compounds tested show different scavenging activity at different rates. At a concentration of 200 μg/mL, compound **A6** (63.11%) showed the highest activity in the anthracene-based ( **A** ) series, while compound **A5** (14.77%) showed the lowest activity. In the pyrene-based ( **P** ) series of compounds, **P6** (20.72%) had the highest activity, while **P1** (4.79%) had the lowest activity. When the two series were compared, it was found that the **A** series compounds showed much better activity than the **P** series compounds. The DMSO solution of the compounds showed less radical scavenging ability than Trolox (99.48%) at the same concentration.

#### 2.2.2. Metal chelating ability

**Figure 2:**
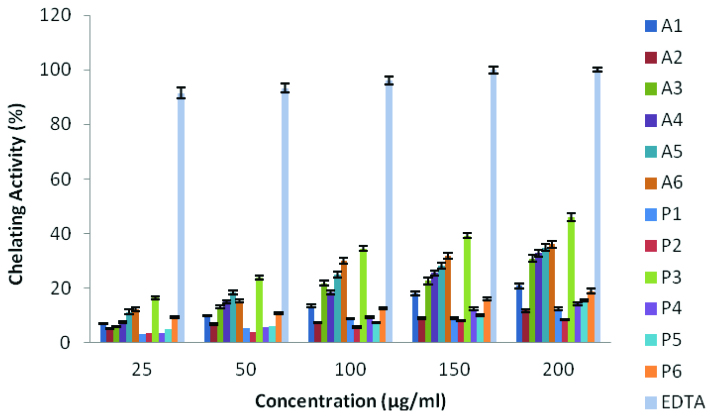
Metal chelating activity of compounds.

The chelating agents can prevent free radical formation by stabilizing the transition metals in living systems, thereby reducing the damage caused by free radicals [35]. Figure 2 shows the chelating activity of compounds. In **A** series, the highest activity was observed in compound A6 and the lowest activity was observed in compound **A2** . In **P** series, the highest activity was obtained for **P3** and the lowest activity was obtained for compound **P2** . When the concentration of solutions of the test compounds increased from 25 μg/mL to 200 μg/mL, the chelating activity increased from 12.2% to 36.1% for **A6** , 5.1% to 11.7% for **A2** , 16.4% to 45.8 for **P3** , 3.3% to 8.2% for **P2** and 91.5% to 100% for EDTA, respectively. EDTA exhibited better activity than compounds at all concentrations studied.

#### 2.2.3. Reducing power ability

**Figure 3:**
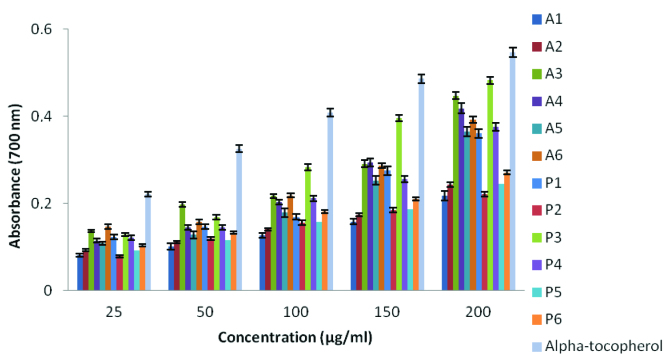
Reducing the power of novel Schiff base derivatives.

The antioxidant capacity of organic or inorganic substances may rely on the reducing power data. Complexes with high reducing power behave as electron donors, thus, they can act as reducing agents for oxidative intermediates in lipid peroxidation process which promotes them to be used as antioxidants [36]. The reducing power of metal complexes was shown in Figure 3. Reducing power of samples increased as the concentration increased and α-tocopherol showed higher activity than the present compounds. Among the Schiff base derivatives, the highest reducing power activity was obtained from **P3** as 0.482 at 200 μg/mL. Moreover, the rest of the series have lower reducing power, such as; **A3** as 0.447, **A4** as 0.418, **A6** as 0.392 and **P4** as 0.375 at the same concentration.

### 2.3. Antimicrobial ability

Three types of gram-positive and 3 types of gram-negative bacteria were considered to investigate the antimicrobial effect of the compounds. The inhibition zones of the compounds studied here against the gram-positive and gram-negative bacteria are listed in Table 2. The results indicate that the compounds formed inhibition zones in the range of 0–16 mm. According to results, compounds **A6** , **P3** and **P6** showed activity against all bacteria tested. On the other hand, **A4** , **A5** , **P4** and **P5** did not showed antibacterial activity against any bacteria. **P3** showed 16, 14 and 13 millimeter inhibition zones against *B. cereus* , *E. coli* and *P. aeriginosa* , respectively. However, standard antibiotics (tetracycline and streptomycin) were found to be more effective than the new compounds tested against bacteria.

**Table 2:** Inhibition zone of the compounds against bacteria.

	Compounds and standard antibiotic discs^a^													
Bacteria	A1	A2	A3	A4	A5	A6	P1	P2	P3	P4	P5	P6	S	TE
*S. aureus*	-	-	10	-	-	12	8	-	11	-	-	12	16	24
*B. cereus*	-	9	8	-	-	13	10	8	16	-	-	14	18	20
*E. hirae*	8	7	8	-	-	12	9	7	11	-	-	13	19	22
*E. coli*	7	-	-	-	-	7	10	7	14	-	-	9	24	23
*P. aeriginosa*	-	-	-	-	-	9	8	-	13	-	-	13	22	14
*L. pneumophila*	8	-	7	-	-	10	7	7	9	-	-	12	15	21

^a^Inhibition diameter in millimeters. S = Streptomycin (10 g), and TE = Tetracycline (30 g).

### 2.4. DNA binding ability

**Figure 4:**
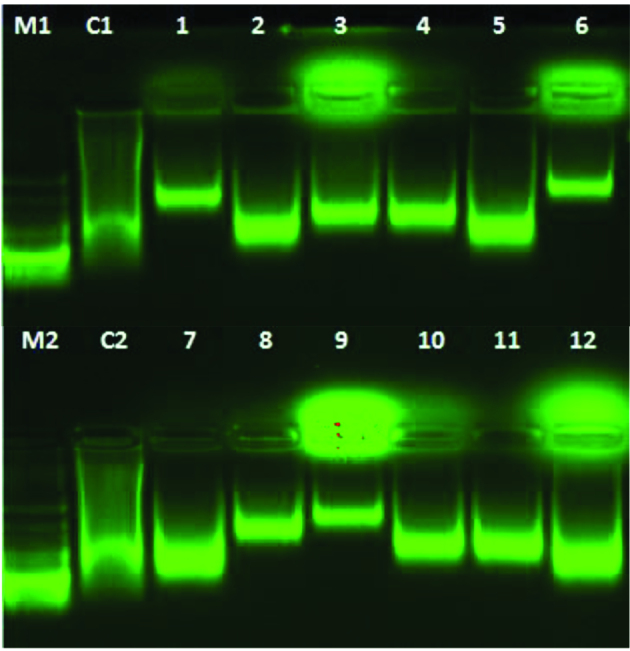
DNA binding of newly synthesized compounds. Lane M1 and M2, DNA Marker; Lane C1 and C2, Control, CT- DNA; Lane 1, CT- DNA + 500 μg/mL of A1; Lane 2, CT- DNA + 500 μg/mL of A2; Lane 3, CT- DNA + 500 μg/mL of A3; Lane 4, CT- DNA + 500 μg/mL of A4; Lane 5, CT- DNA + 500 μg/mL of A5; Lane 6, CT- DNA + 500 μg/mL of A6; Lane 7, CT- DNA + 500 μg/mL of P1; Lane 8, CT- DNA + 500 μg/mL of P2; Lane 9, CT- DNA + 500 μg/mL of P3; Lane 10, CT- DNA + 500 μg/mL of P4; Lane 11, CT- DNA + 500 μg/mL of P5; Lane 12, CTDNA + 500 μg/mL of P6.

In agarose gel electrophoresis method, molecules act according to their mass, charge and shape. As a result of binding of the compounds to CT-DNA, the free DNA moves faster on the gel because it is smaller than the bound DNA [37]. Agarose gel electrophoresis results of compounds are given in Figure 4. According to the results, **A6** and **P3** compounds had the highest DNA binding capacity and a fragment of the DNA did not move well from the agarose gel. **A2** , **A5** , **P4** and **P5** compounds were found to have the lowest binding activity.

### 2.5. Computational calculations

**Figure 5:**
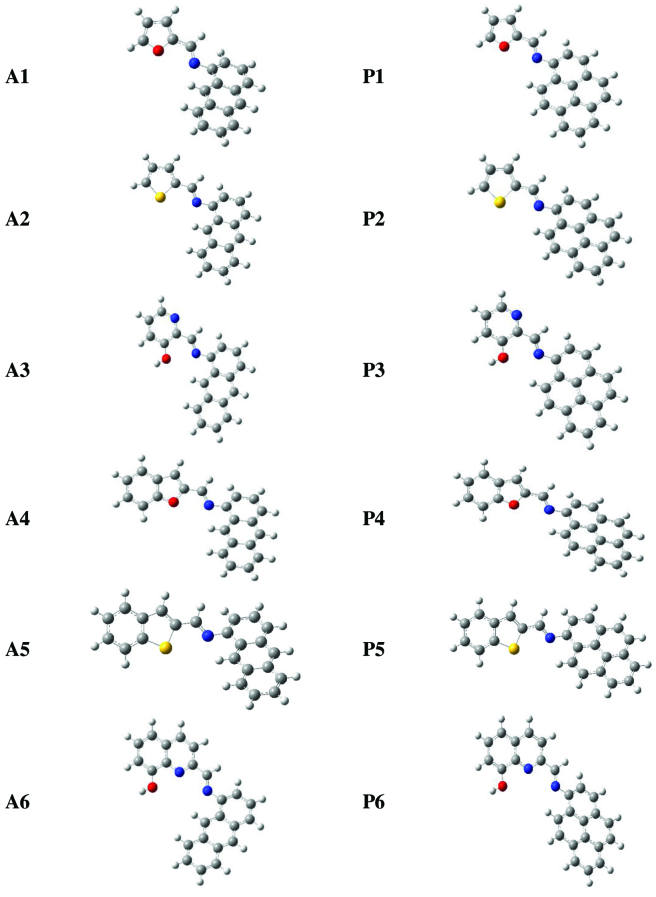
Geometry optimized structures for A and P series.

Density functional theory at B3LYP/6-311++G(d,p) level was applied to get 3-dimensional optimized geometries of **A** and **P** series (Figure 5) with no symmetry restrictions. Bond lengths, angles and dihedral angles were computed by the same method to obtain the structural parameters. All theoretical calculations were performed by Gaussian 16W [38] package program. Then, vibrational analyses on the Schiff base derivatives and their metal complexes were carried on by the application of the aforementioned computational method employed for geometry optimizations. The structures of all systems were located at a minimum on the potential energy surface since the outputs of the frequency computations did not possess any imaginary data.

The geometry optimized structures of the Schiff bases can be seen in Figure 5. Although most of the structures are planar, in some cases anthracene and pyrene moieties are tilted out of plane. Combination of heteroaromatic systems with fluorescent abilities of anthracene and pyrene would potentially create very important metal sensor compounds. Therefore, present systems were investigated in terms of metal coordination capability. Thereafter the optimized geometries obtained and confirmed to be at least a minimum energy structure, vertical transition energies of **A** and **P** series and their metal complexes were computed with time-dependent density functional theory (TD-DFT). The absorption spectra and oscillator strengths were obtained by the calculation of singlet transitions from the optimized ground state to the excited states by using TD-DFT with B3LYP/6-311++G(d,p) basis set [39]. Upon application of TD-DFT method we obtained the electronic absorption spectra, including maximum absorption wavelengths.

**Figure 6:**
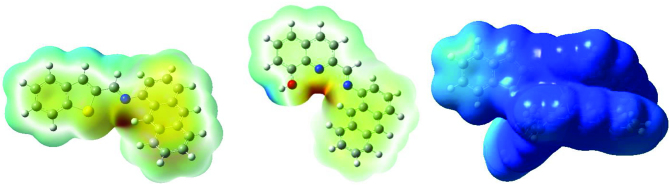
Three-dimensional electrostatic potential maps for A6 and A5_Zn.

In Figure 6, 3-dimensional electrostatic potential maps obtained as a result of single point calculations performed on geometry optimized structures ( **A5** and **A6** ) are shown. These maps show how electrons ar dispersed on structures. Red zones are electron-dense (negative), green zones are electron-poor and blue zones are very poor (positive).

When the electrostatic potential maps of the structures are examined, we see that the region where coordination is expected is rich in electron. Therefore, the potential of cations to approach this region and form a coordination complex is high. Following metal coordination, it was seen that the electron distribution in the structure was more positive with the dispersion of +2 charge from the metal ion. The direct effect of metal insertion on the electronic structure is a proof of metal coordination.

**Figure 7:**
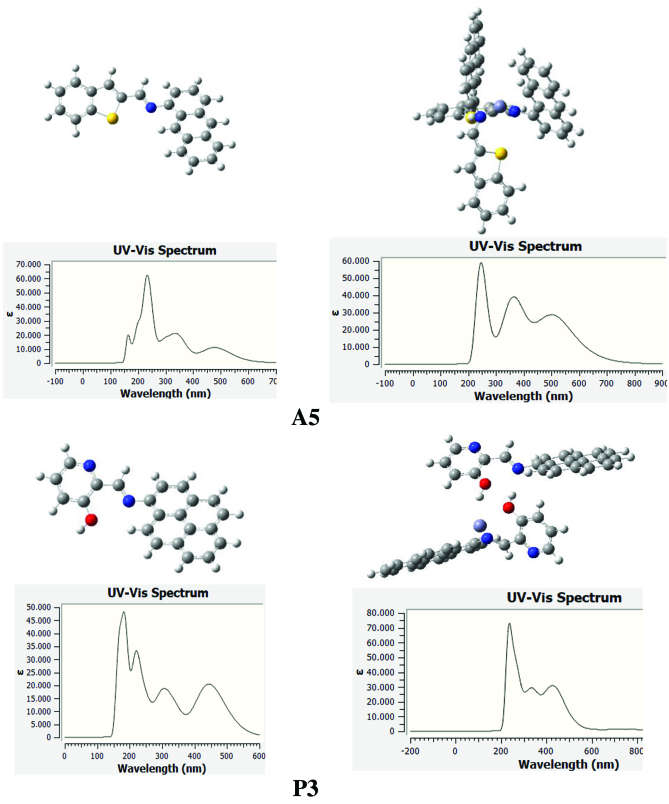
Absorption spectra for A5, P3, A5_Zn and P3_Zn.

Metal coordination resulted in changes both in the electronic properties and the structure of the parent system completely. One way to observe this change and prove coordination is to calculate the absorption spectra by applying TD-DFT. In this study, B3LYP/6-311++G(d,p) computational method was used for TDDFT applications. One hundred excitation levels were taken into account for the acquisition of UV-VIS spectra. The absorption spectrum of the **A5** structure has three sharp bands at 220 nm, 340 nm and 500 nm (Figure 7). In addition, a weak band was observed at 180 nm. When **A5** is coordinated with Zn^2+^, wide bands are replaced by sharp bands in the absorption spectrum of the molecule. Also, blue shifting is observed in the bands. Literature research shows that metal is coordinated with 2 structures in order to keep the intramolecular planarity intact. In this case, the central metal cation was complexed with 2 **A5** compounds. As seen in Figure 7, anthracenes are perpendicular to each other and have not lost their flatness. In the spectrum of this structure obtained, it was observed that the bands became sharp and the small band at 180 nm disappeared. A similar behavior has been observed in the case of **P3** and **P3_Zn** complex.

## 3. Experimental

### 3.1. General

Predried glassware was used to carry out all experiments in argon atmosphere. Chemicals for in the biological assay studies were bought from Sigma-Aldrich Chemie GmbH (Sternheim, Germany). The blank antimicrobial test discs were obtained from Oxoid (7.0 mm, Oxoid Ltd, Basingstoke, Hants, UK). ^1^HNMR and 13 CNMR spectra were measured in CDCl_3_ on an Agilent NMR spectrometer (400 MHz). ^1^H (400 MHz) and 13 CNMR (100 MHz). Chemical shifts are expressed in ppm relative to CDCl_3_ (δ 7.26 and 77.0 for ^1^H and ^13^CNMR, respectively). Thermo Scientific Q Exactive instrument (Thermo Fisher Scientific Inc., Waltham, MA, USA) was used to get mass spectra for the final products. Melting point data was obtained from Stuart SMP3 instrument.

For separation and purification flash column chromatography was practiced through silica gel (60-mesh; Merck KGaA, Darmstadt, Germany) in thick-walled glass columns. The monitoring of the reactions was performed by thin-layer chromatography (TLC) using analytical aluminum plates with brand Merck 0.2-m silica gel 60 F254 and visualization was done with UV-lamp. Anhydrous magnesium sulfate was used to dry over all the extracts and solvents were removed under low temperature and reduced pressure by using a Heidolph brand rotary evaporator.

### 3.2. Synthesis of Schiff base derivatives, A1-A6 and P1-P6

All Schiff bases were synthesized according to the same procedure. 1-aminoanthracene or 1-aminopyrene (1.0 mmol) and aldehyde (1.0 mmol) were dissolved in 5 mL ethanol. Acetic acid (2–3 drops) was added to the mixture and stirred overnight at room temperature. The solid product was filtered, washed several times wit ethanol and dried in vacuum.

#### 3.2.1. (E)-N-(furan-2-ylmethylene)anthracen-1-amine, A1

Yellow solid. mp:124–126 °C. IR ν_max_ (neat cm^-1^) : 3043, 2980, 1927, 1675, 1613, 1551, 1453, 1391, 1017, 881, 751, 731. ^1^H NMR (CDCl3 , 400 MHz): δ 8.99 (s, 1H), 8.41 (d, J = 7.3 Hz, 2H), 8.10–8.08 (m, 1H), 8.02–8.00 (m, 1H), 7.88 (d, J = 8.6 Hz, 1H), 7.70 (s, 1H), 7.50–7.47 (m, 2H), 7.44–7.40 (m, 1H), 7.05–7.04 (m, 1H), 6.99–6.97 (m, 1H), 6.60–6.59 (m, 1H); 13 C NMR (CDCl3 , 100 MHz): δ 152.4, 149.6, 148.0, 145.8, 132.1, 132.0, 131.6, 128.8, 128.0, 127.7, 126.4, 126.0, 125.7, 125.4, 125.3, 123.1, 116.4, 112.3, 111.3. LC-MS/MS. Anal. Calcd for C_19_H_13_NO [M+H]^+^: m/z272.1070. Found: m/z272.1077.

#### 3.2.3. (E)-2-((anthracen-1-ylimino)methyl)pyridin-3-ol, A3

Yellow solid. mp: 105–107 °C. IR ν_max_ (neat cm^−1^): 3046, 2980, 1913, 1614, 1591, 1449, 1341, 1176, 1016, 865, 801, 717. ^1^H NMR (CDCl3 , 400 MHz): δ 13.44 (s, 1H), 9.01 (s, 1H), 8.76 (s, 1H), 8.46 (s, 1H), 8.34 (dd, J = 1.4 and 4.4 Hz, 1H), 8.07–8.05 (m, 1H), 8.02–8.00 (m, 1H), 7.97 (d, J = 8.6 Hz, 1H), 7.51–7.48 (m, 3H), 7.47–7.46 (m, 1H), 7.36 (dd, J = 4.4 and 8.4 Hz, 1H), 7.22 (dd, J = 0.7 and 7.0 Hz, 1H); 13 C NMR (CDCl3 , 100 MHz): δ 164.6, 158.5, 145.5, 141.5, 137.6, 132.0, 131.9, 131.9, 128.7, 128.1, 128.0, 126.9, 126.8, 126.5, 126.0, 125.8, 125.1, 125.0, 121.9, 113.2. LC-MS/MS. Anal. Calcd for C_20_H_14_N_2_O [M+H]^+^: m/z299.1179. Found: m/z299.1190.

#### 3.2.4. (E)-N-(benzofuran-2-ylmethylene)anthracen-1-amine, A4

Yellow solid. mp: 142–144 °C. IR ν_max_ (neat cm^-1^): 3049, 2980, 2888, 1923, 1614, 1549, 1449, 1305, 1125, 948, 877, 734. ^1^H NMR (CDCl_3_, 400 MHz): δ 8.98 (s, 1H), 8.58 (s, 1H), 8.44 (s, 1H), 8.09–8.07 (m, 1H), 8.03–8.00 (m, 1H), 7.93–7.91 (m, 1H), 7.73–7.72 (m, 1H), 7.72–7.71 (m, 1H), 7.50–7.45 (m, 4H), 7.37 (d, J = 0.8 Hz, 1H), 7.36–7.32 (m, 1H), 7.05 (dd, J = 0.8 and 7.0 Hz, 1H); 13 C NMR (CDCl_3_, 100 MHz): δ 156.0, 153.3, 149.4, 148.6, 132.1, 132.0, 131.6, 128.8, 127.9, 127.8, 127.5, 127.2, 126.8, 126.0, 125.7, 125.4, 125.3, 123.6, 123.1, 122.3, 113.2, 112.6, 111.4. LC-MS/MS. Anal. Calcd for C_23_H_15_NO [M+H]^+^: m/z322.1226. Found: m/z322.1237.

#### 3.2.5. (E)-N-(benzo[b]thiophen-2-ylmethylene)anthracen-1-amine, A5

Yellow solid. mp: 171–173 °C. IR ν_max_ (neat cm^-1^): 3048, 1914, 1602, 1587, 1474, 1310, 1130, 1016, 866, 810, 727. ^1^H NMR (CDC_13_, 400 MHz): δ 8.96 (s, 1H), 8.81 (s, 1H), 8.43 (s, 1H), 8.10–8.08 (m, 1H), 8.03–8.00 (m, 1H), 7.96–7.85 (m, 3H), 7.75 (s, 1H), 7.50–7.39 (m, 5H), 7.07 (dd, J = 0.8 and 7.0 Hz, 1H); ^13^C NMR (CDC_l3_, 100 MHz): δ 153.4, 148.7, 143.3, 141.4, 139.4, 132.1, 132.0, 131.6, 129.6, 128.9, 127.9, 127.8, 126.7, 126.6, 126.0, 125.7, 125.4, 125.3, 124.9, 124.8, 123.0, 122.9, 111.4. LC-MS/MS. Anal. Calcd for C_23_H_15_NS [M+H]^+^: m/z338.0998. Found: m/z338.1008.

#### 3.2.6. (E)-2-((anthracen-1-ylimino)methyl)quinolin-8-ol, A6

Yellow solid. mp: 140–142 °C. IR ν_max_ (neat cm^−1^): 3413, 3040, 1917, 1613, 1565, 1469, 1454, 1230, 1087, 874, 839, 726. ^1^H NMR (CDC_l3_, 400 MHz): δ 8.96 (s, 1H), 8.90 (s, 1H), 8.63 (d, J = 8.6 Hz, 1H), 8.46 (s, 1H), 8.33 (d, J = 8.6 Hz, 1H), 8.05–8.02 (m, 2H), 7.95 (d, J = 8.8 Hz, 1H), 7.57–7.42 (m, 6H), 7.27–7.25 (m, 1H), 7.16 (dd, J = 0.7 and 7.0 Hz, 1H); ^13^C NMR (CDCl_3_, 100 MHz): δ 160.2, 152.6, 148.4, 137.9, 136.7, 132.1, 132.0, 131.7, 129.3, 129.2, 128.7, 128.0, 128.0, 127.7, 127.3, 126.2, 125.7, 125.5, 125.3, 122.7, 119.6, 118.0, 111.5, 110.7. LC-MS/MS. Anal. Calcd for C_24_H_16_N_2_O [M+H]^+^: m/z349.1335. Found: m/z349.1348.

#### 3.2.7. (E)-N-(furan-2-ylmethylene)pyren-1-amine, P1

Yellow solid. mp: 107–109 °C. IR ν_max_ (neat cm^−1^): 3120, 3041, 2980, 1912, 1630, 1616, 1590, 1478, 1273, 1020, 839, 804, 715, 679, 592. ^1^H NMR (CDCl_3_, 400 MHz): δ 8.69 (d, J = 9.2 Hz, 1H), 8.49 (s, 1H), 8.18–8.10 (m, 4H), 8.02–7.99 (m, 3H), 7.71 (d, J = 1.7 Hz, 1H), 7.67 (d, J = 8.1 Hz, 1H), 7.08 (dd, J = 0.5 and 3.5 Hz, 1H), 6.62 (dd, J = 1.7 and 3.5 Hz, 1H); ^13^C NMR (CDCl_3_, 100 MHz): δ 152.7, 148.4, 145.8, 145.5, 131.6, 131.5, 129.7, 127.2, 127.1, 126.7, 126.1, 125.5, 125.4, 125.2, 125.0, 124.9, 124.8, 123.4, 116.2, 115.2, 112.3. LC-MS/MS. Anal. Calcd for C_21_H_13_NO [M+H]^+^: m/z296.1070. Found: m/z296.1080.

#### 3.2.8. (E)-N-(thiophen-2-ylmethylene)pyren-1-amine, P2

Yellow solid. mp: 130–132 °C. IR ν_max_ (neat cm^−1^): 3081, 3042, 1919, 1626, 1583, 1492, 1422, 1206, 961, 839, 726, 679. ^1^H NMR (CDCl_3_, 400 MHz): δ 8.74 (s, 1H), 8.71 (d, J = 9.2 Hz, 1H), 8.19–8.15 (m, 2H), 8.13–8.09 (m, 2H), 8.02–8.00 (m, 3H), 7.68 (d, J = 8.1 Hz, 1H), 7.58–7.56 (m, 1H), 7.52 (dd, J = 1.1 and 3.6 Hz, 1H), 7.17 (dd, J = 3.6 and 5.0 Hz, 1H); ^13^C NMR (CDCl_3_, 100 MHz): δ 153.2, 144.9, 143.6, 132.2, 131.6, 131.5, 130.5, 129.7, 127.9, 127.3, 127.1, 126.7, 126.1, 125.6, 125.5, 125.3, 125.0, 124.9, 124.8, 123.4, 115.3. LC-MS/MS. Anal. Calcd for C_21_H_13_NS [M+H]^+^: m/z312.0841. Found: m/z312.0848.

#### 3.2.9. (E)-2-((pyren-1-ylimino)methyl)pyridin-3-ol, P3

Yellow solid. mp: 200–202 °C. IR ν_max_ (neat cm^−1^): 3044, 2980, 1912, 1627, 1606, 1470, 1445, 1293, 1169, 976, 831, 708. ^1^H NMR (CDCl_3_, 400 MHz): δ 13.69 (bs, 1H), 9.14 (s, 1H), 8.50 (d, J = 9.2 Hz, 1H), 8.37 (d, J = 4.4 Hz, 1H), 8.23–8.20 (m, 3H), 8.16 (d, J = 9.2 Hz, 1H), 8.08–8.03 (m, 3H), 7.92–7.90 (d, J = 8.2 Hz, 1H), 7.50 (d, J = 8.4 Hz, 1H), 7.38 (dd, J = 4.4 and 8.4 Hz, 1H); 13 C NMR (CDCl_3_, 100 MHz): δ 164.4, 158.5, 141.5, 141.1, 137.8, 131.4, 131.2, 130.9, 128.4, 127.7, 127.2, 126.8, 126.4, 125.8, 125.8, 125.7, 125.6, 125.3, 125.1, 124.6, 122.0, 115.6. LC-MS/MS. Anal. Calcd for C_22_H_14_NO [M+H]^+^: m/z323.1179. Found: m/z323.1189.

#### 3.2.10. (E)-N-(benzofuran-2-ylmethylene)pyren-1-amine, P4

Yellow solid. mp: 157–159 °C. IR ν_max_ (neat cm^−1^): 3043, 2878, 1925, 1629, 1590, 1292, 1129, 954, 828, 749, 716. ^1^H NMR (CDCl_3_, 400 MHz): δ 8.75 (d, J = 9.2 Hz, 1H), 8.63 (s, 1H), 8.20–8.13 (m, 4H), 8.03 (s, 2H) 8.02–7.99 (m, 1H), 7.75–7.70 (m, 3H), 7.49–7.45 (m, 1H), 7.36–7.31 (m, 2H); ^13^ C NMR (CDCl_3_, 100 MHz) δ 156.0, 153.6, 148.8, 145.1, 131.5, 131.4, 130.1, 127.9, 127.3, 127.2, 127.1, 127.0, 126.2, 125.6, 125.5, 125.2, 125.1, 125.0, 124.8, 123.6, 123.4, 122.3, 115.1, 113.0, 112.2. LC-MS/MS. Anal. Calcd for C_25_H_15_NO [M+H]^+^: m/z346.1226. Found: m/z346.1238.

#### 3.2.11. (E)-N-(benzo[b]thiophen-2-ylmethylene)pyren-1-amine, P5

Yellow solid. mp: 173–175 °C. IR ν_max_ (neat cm^−1^): 3046, 1917, 1626, 1605, 1529, 1433, 1288, 838, 727, 679, 619. ^1^H NMR (CDCl_3_, 400 MHz): δ 8.90 (s, 1H), 8.75 (d, J = 9.2 Hz, 1H), 8.20–8.12 (m, 4H), 8.03–7.99 (m, 3H), 7.94–7.92 (m, 1H), 7.86–7.84 (m, 1H), 7.77 (d, J = 8.2 Hz, 1H), 7.75 (s, 1H), 7.47–7.39 (m, 2H); ^13^C NMR (CDCl_3_, 100 MHz): δ 153.4, 144.3, 143.8, 141.3, 139.5, 131.5, 131.4, 130.0, 129.5, 127.3, 127.2, 127.0, 126.5, 126.1, 125.9, 125.5, 125.3, 125.1, 125.0, 124.9, 124.8, 124.7, 123.4, 122.9, 115.1. LC-MS/MS. Anal. Calcd for C_25_H_15_NS [M+H]^+^: m/z362.0998. Found: m/z362.1010.

#### 3.2.12. (E)-2-((pyren-1-ylimino)methyl)quinolin-8-ol, P6

Yellow solid. mp: 210–212 °C. IR ν_max_ (neat cm^−1^): 3380, 3037, 1916, 1629, 1596, 1507, 1467, 1234, 1187, 1084, 963, 838, 752, 718, 585. ^1^H NMR (CDCl_3_, 400 MHz): δ 9.68 (bs, 1H), 9.06 (s, 1H), 8.72 (d, J = 9.2 Hz, 1H), 8.56 (d, J = 8.6 Hz, 1H), 8.37 (d, J = 8.6 Hz, 1H), 8.25 (d, J = 8.2 Hz, 1H), 8.21–8.18 (m, 2H), 8.14 (d, J = 9.2 Hz, 1H), 8.06 (d, J = 4.3 Hz, 2H), 8.03–8.00 (m, 1H), 7.93 (d, J = 8.2 Hz, 1H), 7.48–7.39 (m, 2H), 7.15 (dd, J = 1.2 and 7.5 Hz, 1H); ^13^C NMR (CDCl_3_, 100 MHz): δ 161.2, 153.9, 152.9, 144.0, 138.6, 137.0, 131.4, 131.2, 130.3, 129.8, 129.3, 127.6, 127.5, 127.4, 126.6, 126.1, 125.9, 125.6, 125.5, 125.0, 124.5, 123.2, 119.1, 118.1, 115.1, 112.1. LC-MS/MS. Anal. Calcd for C_26_H_16_N_2_O [M+H]^+^: m/z373.1335. Found: m/z373.1349.

### 3.3. Free radical scavenging ability

Compounds were prepared with concentrations of 25, 50, 100, 150, and 200 μg/mL in DMSO. Into different concentrations of 0.5 mL of the compounds, 2.0 mL of methanol DPPH solution was added, and then, this mixture of solutions were incubated at room temperature in the dark for 30 min. Using an absorbance spectrophotometer the absorbance data at 517 nm were measured. Positive control for the measurement was performed through Trolox [40].

### 3.4. Metal chelating ability

The metal chelating property of the compounds was performed by following the method described in the literature by Dundar et al. [41]. The compounds of different concentrations (25–200 /g/mL) in 0.5 mL DMSO were prepared. To the compounds were added 1.85 mL of methanol followed by 0.1 mL of 2 mmol/L of iron chloride. Addition of ferrozine (0.2 mL of 5 mmol/L) to the solution mixture started the reaction. At last, the absorbance was measured at 562 nm, after the mixture was incubated at room temperature for 10 min. The results were given in terms of percent inhibition of ferrozine-Fe^2+^ complex formation. Positive control was done using EDTA.

### 3.5. Power of reducing ability

The method reported by Oyaizu [42] was applied in order to determine the reducing power activities of the present compounds. Compounds of different concentrations (25–200 /g / mL) in 0.25 mL DMSO were prepared. Into the solutions of the compounds, 0.25 mL of 200 mM sodium phosphate buffer (pH 6.6) and 0.25 mL of 1.25 mL of 1.0% potassium ferricyanide were added, respectively. Incubation of the mixture at 50 °C for 30 min followed by addition of 0.25 mL of 10% trichloroacetic acid. The solution was then centrifuged at 1000 rpm fo 10 min. After that, the supernatant fluid of the solution (0.5 mL) was mixed with 0.5 mL deionised water and 0.2 mL ferric chloride (0.1%). Finally, the absorbance data of the solution was measured at 700 nm against a blank. The results were compared through the standard, α-tocopherol.

### 3.6. Antibacterial ability

Disk diffusion method was employed to test the antibacterial activity of the compounds [43]. Gram– bacteria, *Pseudomonas aeruginosa* (ATCC 9027), *Legionella pneumophila* subsp. *pneumophiia* (ATCC 33152) and *Escherichia coli* (ATCC 10536) and gram+ bacteria, *Enterococcus hirae* (ATCC 10541), *Staphylococcus aureus* (ATCC 6538) and *Bacillus cereus* were used to determine the antibacterial activity of the present compounds. Sterile antibiotic discs were impregnated with 20 μL of compound and the discs were placed in a nutrient agar poured petri dish inoculated with bacteria. As the negative control, a disc impregnated with DMSO and as the positive control tetracycline (30 μg) and streptomycin (10 μg) discs were used, respectively. The cultures of the bacteria were incubated for 24 h at 37 °C, which was followed by measuring the inhibition zone diameters in mm to determine the antibacterial ability of the compounds.

### 3.7. DNA binding ability

To determine the DNA binding activity of the compounds, CT-DNA (conc: 20 μg/mL) was diluted with sterile deionized water (1:5 ratio). 4 μL of this DNA solution was mixed with 6 μL of the present compounds in DMSO (500 μg/mL). The mixed solutions were incubated at 37 °C for 8 h in the dark and after incubation time 3 μL DNA loading dye was added. Loading of the samples were performed on a gel containing 8 μL 0.05% ethidium bromide. 80 V in TAE buffer (50 mM Tris base, 50 mM acetic acid, 2 mM EDTA, pH: 7.8) was used for electrophoresis application for 50 min. The resultant gel was viewed and photographed under UV radiation [44].

## 4. Conclusion

As a result, the design and synthesis of novel anthracene- and pyrene-based Schiff base derivatives were described Heteroaromatic aldehydes were reacted with aminoanthracene and aminopyrene, seperately and novel Schiff base derivatives **A1-A6** and **P1-P6** were obtained in very good yields. Free radical scavenging, metal chelating, reducing power, antibacterial and DNA binding ability of newly synthesized compounds were tested. **A** series of compounds showed much better radical scavenging activity than the **P** series compounds. The compound **P3** demonstrated highest metal chelating activity. In 3 antioxidant test systems, the standarts exhibited higher antioxidant activities than the DMSO solution of compounds. In addition, **P3** and **A6** showed antibacterial activity against the bacteria employed and both compounds were very well bound to CT-DNA. The outcomes of this study indicate that, newly studied compounds have DNA binding activity and can be a potential drug candidates for cancer treatment with further modification in their structure in the future.

## References

[1] (2018). FRET between riboflavin and 9-Anthraldehyde based fluorescent organic nanoparticles possessing antibacterial activity. Journal of Fluorescence.

[2] (2018). Selective detection of Co$^{2+}$ by fluorescent nano probe: diagnostic approach for analysis of environmental samples and biological activities. Spectrochima Acta A.

[3] (2007). Synthesis, characterization and antibacterial activity of azomethine derivatives derived from 2-formylphenoxyacetic acid. Molecules.

[4] (2010). Synthesis, antibacterial and anti-fungal activities of some novel schiff bases containing 2,4-disubstituted thiazole ring. Eurpean Journal Medicinal Chemistry.

[5] (2014). Schiff's base derivatives bearing nitroimidazole and quinoline nuclei: new class of anticancer agents and potential EGFR tyrosine kinase inhibitors. Bioorganic Medicinal Chemistry Letters.

[6] (2011). Synthesis, biological assay in vitro and molecular docking studies of new Schiff base derivatives as potential urease inhibitors. Eurpean Journal Medicinal Chemistry.

[7] (2013). Synthesis, evaluation of antioxidant activity and crystal structure of 2,4-dimethylbenzoylhydrazones. Molecules.

[8] (2013). Antioxidant properties of phenolic Schiff bases: structure-activity relationship and mechanism of action. Journal Computater-Aided Molecular Design.

[9] (2014). Synthesis of 4-methoxybenzoylhydrazones and evaluation of their antiglycation activity. Molecules.

[10] (2015). Schiff's bases of quinazolinone derivatives: synthesis and SAR studies of a novel series of potential anti-inflammatory and antioxidants. Bioorganic Medicinal Chemistry Letters.

[11] (2010). New di- and triorganotin (IV) complexes of tripodal Schiff base ligand containing three imidazole arms: synthesis, structural characterization, anti-inflammatory activity and thermal studies. Journal of Organometallic Chemistry.

[12] (2014). Synthesis and antitumor activities of novel hybrid molecules moiety. Bioorganic Medicinal Chemistry Letters.

[13] (2017). Binary and ternary copper (II) complexes of a new Schiff base ligand spectral, thermal, antimicrobial and antitumor studies. Journal of Molecular Structure.

[14] (2018). -d] pyrimidine derivatives containing a Schiff base moiety as potential antiviral agents. Pyrazolo[3.

[15] (2017). Synthesis and biological evaluation of schiff bases of 4-aminophenazone as an antiinflammatory, analgesic and antipyretic agent. Journal of Saudi Chemical Society.

[16] (2012). Inhibition of HIV-1 nuclear import via schiff base formation with arylene bis(methylketone). Compounds.

[17] (2014). Synthesis, interaction with DNA and antiproliferative activities of two novel Cu (II) complexes with Schiff base of benzimidazole. Sensors and Actuators A.

[18] (2013). Synthesis and antiproliferative activity of some new fluorinated Schiff bases derived from 1, 2, 4-triazoles. Journal of Fluorine Chemistry.

[19] (2018). Discriminating chemosensor for detection of Fe3$+$in aqueous media by fluorescence quenching methodology. Bulletin of Korean Chemical Society.

[20] (2018). Intracellular imaging of zinc ion in living cells by fluorescein based organic nanoparticles. Sensors and Actuators B.

[21] (2008). A new trend in rhodamine-based chemosensors: application of spirolactam ringopening to sensing ions. Chemical Society Reviews.

[22] (2016). Synthesis, characterization, antimicrobial screening and computational studies of 4-3-(4-methoxy-. - phenyl-1.

[23] (2011). Schiff bases: a short review of their antimicrobial activities. Journal of Advanced Research.

[24] (2010). Antitumour activities of some schiff bases derived from benzoin, salicylaldehyde, amino phenol and 2, 4 dinitrophenylhydrazine. Thai Journal of Pharmaceutical Sciences.

[25] (2013). In vivo anticancer and histopathology studies of Schiff bases on Ehrlich ascitic carcinoma cells: 1st cancer update. Arabian Journal of Chemistry.

[26] (2015). Synthesis and anti-proliferative activity of novel azazerumbone conjugates with chalcones. Bioorganic Medicinal Chemistry Letters.

[27] (2013). Extremely fast and highly selective detection of nitroaromatic explosive vapours using fluorescent polymer thin films. Chemical Communications.

[28] (2010). Chemically assembled monolayers of fluorophores as chemical sensing materials. Chemical Society Reviews.

[29] (2018). Chemically diverse small molecule fluorescent chemosensors for copper ion. Coordination Chemistry Reviews.

[30] (2013). Electron rich supramolecular polymers as fluorescent sensors for nitroaromatics. RSC Advances.

[31] (2013). Conformation induced discrimination between picric acid and nitro derivatives/anions with a Cu-pyrene array: the first decision making photonic device. RSC Advances.

[32] (2003). Stereoselective synthesis of $\beta $-lactams with polyaromatic imines: entry to new and novel anticancer agents. Journal of Medicinal Chemistry.

[33] (2016). A reversible pyrene-based turn-on luminescent chemosensor for selective detection of Fe$^{3+}$ in aqueous environment with logic gate application. Journal of Fluorescence.

[34] (2018). Synthesis and biological evaluation of quinoline-triazole and quinolone-triazole conjugates. Turkish Journal of Chemistry.

[35] (2003). Studies on antioxidant activities of mucuna seed (\textit{Mucuna pruriens} var. utilis) extracts and certain non-protein amino/imino acids through in vitro models. Journal of the Science of Food and Agriculture.

[36] (2017). Extraction of antioxidant and antiproliferative ingredients from fruits of Rubus chingii Hu by active tracking guidance. Medicinal Chemistry Communications.

[37] (2018). Synthesis of 2-substituted 8-propargyloxyquinoline derivatives and determination of their antioxidant, antibacterial and DNA binding activities. Turkish Journal of Chemistry.

[38] (2016). Revision B01. Gaussian16.

[39] (1998). Molecular excitation energies to high-lying bound states from timedependent densityfunctional response theory: characterization and correction of the time-dependent local density approximation ionization threshold. Journal of Chemical Physics.

[40] (1958). Antioxidant determinations by the use of a stable free radical. Nature.

[41] (2013). Antioxidant properties of cultured mycelia from four Pleurotus Species produced in submerged medium. International Journal of Food Properties.

[42] (1986). Studies on product of browning reaction prepared from glucose amine. The Japanese Journal of Nutrition and Dietetics.

[43] (2012). Synthesis and in vitro antibacterial activity of novel steroidal (6R)-spiro-. Journal of Heterocyclic Chemistry.

[44] (2015). Synthesis of two new N$_{2}$O$_{4}$ macroacyclic Schiff base ligands and their mononuclear complexes: Spectral, X-ray crystal structural, antibacterial and DNA cleavage activity. Polyhedron.

